# 2,3-Bis(furan-2-yl)pyrazino­[2,3-*f*][1,10]phenanthroline

**DOI:** 10.1107/S160053681302641X

**Published:** 2013-10-05

**Authors:** Wen-xian Dong, Rong-rong Tong, Chang-ge Zheng

**Affiliations:** a School of Chemical and Material Engineering, Jiangnan University, 1800 Lihu Road, Wuxi, Jiangsu Province 214122, People’s Republic of China

## Abstract

The mol­ecule of the title compound, C_22_H_12_N_4_O_2_, is located on a twofold rotation axis. The dihedral angle between the furan and pyrazine rings is 34.8 (7)°, and that between the furan rings is 46.92 (7)°. A π–π stacking interaction occurs between adjacent pyrazino[2,3-*f*][1,10]phenanthroline units, with an interplanar distance of 3.5862 (12) Å.

## Related literature

For the properties of 2,3-dithienyl­pyrazino­[2,3-*f*][1,10]phenanthroline, see: Bencini *et al.* (1999 ▶); Li *et al.* (2010 ▶). For the structure of 2,3-bis­(thio­phen-2-yl)pyrazino­[2,3-*f*][1,10]phenanthroline, see: Zheng *et al.* (2012 ▶) and for the structure of 3-carb­oxy­pyrazino­[2,3-*f*][1,10]-phenanthrolin-9-ium-2-carb­oxyl­ate see: Zhang *et al.* (2010 ▶).
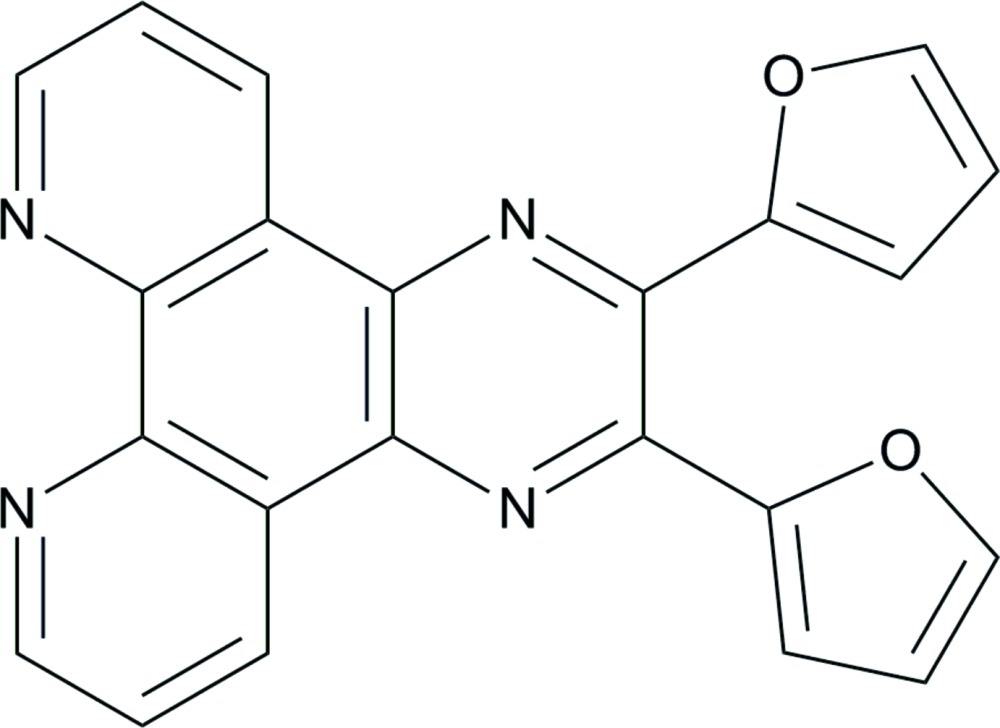


## Experimental

### 

#### Crystal data

C_22_H_12_N_4_O_2_
*M* *_r_* = 364.36
Orthorhombic, 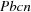

*a* = 7.0994 (14) Å
*b* = 25.014 (5) Å
*c* = 9.4083 (19) Å
*V* = 1670.8 (6) Å^3^
*Z* = 4
Mo *K*α radiation
μ = 0.10 mm^−1^
*T* = 293 K
0.26 × 0.21 × 0.18 mm

#### Data collection

Bruker APEXII CCD diffractometer
Absorption correction: multi-scan (*SADABS*; Sheldrick, 2008*a* ▶) *T*_min_ = 0.975, *T*_max_ = 0.983
7791 measured reflections
1565 independent reflections
1382 reflections with *I* > 2σ(*I*)
*R*_int_ = 0.035

#### Refinement

*R*[*F* ^2^ > 2σ(*F* ^2^)] = 0.051
*wR*(*F* ^2^) = 0.114
*S* = 1.18
1565 reflections
127 parameters
H-atom parameters constrained
Δρ_max_ = 0.16 e Å^−3^
Δρ_min_ = −0.19 e Å^−3^

### 

Data collection: *APEX2* (Bruker, 2005 ▶); cell refinement: *SAINT* (Bruker, 2005 ▶); data reduction: *SAINT*; program(s) used to solve structure: *SHELXS97* (Sheldrick, 2008*b* ▶); program(s) used to refine structure: *SHELXL97* (Sheldrick, 2008*b* ▶); molecular graphics: *SHELXTL* (Sheldrick, 2008*b* ▶); software used to prepare material for publication: *SHELXTL*.

## Supplementary Material

Crystal structure: contains datablock(s) I, New_Global_Publ_Block. DOI: 10.1107/S160053681302641X/bt6933sup1.cif

Structure factors: contains datablock(s) I. DOI: 10.1107/S160053681302641X/bt6933Isup2.hkl

Supplementary material file. DOI: 10.1107/S160053681302641X/bt6933Isup3.cml

Additional supplementary materials:  crystallographic information; 3D view; checkCIF report

## Figures and Tables

**Fig. 1.**
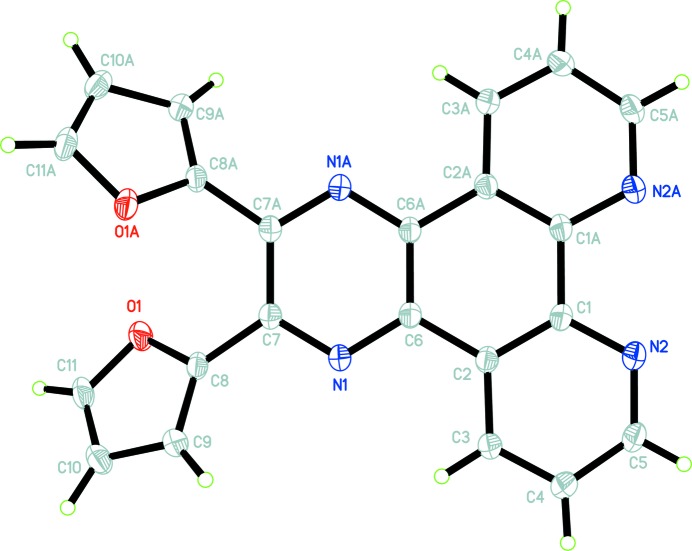
The asymmetric unit of the title complex showing 30% probability displacement ellipsoids and the atom-numbering scheme.

## supplementary crystallographic information

### 1. Comment

2,3-Di(furan-2-yl)pyrazino[2,3-*f*][1,10]phenanthroline as a ligand has been extensively used as ligand in both analytical and preparative coordination chemistry (Bencini *et al.*, 1999; Li *et al.* 2010), due to its rigid structure and fluorescence property.

The structure of 2,3-di(furan-2-yl)pyrazino[2,3-*f*][1,10]phenanthroline, C_22_H_12_N_4_O_2_, has orthorhombic (*Pbcn*) symmetry. The dihedral angles between the furan ring and the pyrazine ring is 34.77 (67)°.

### 2. Experimental

The title compound was synthesized by using 1,10-phenanthroline and 2-furaldehyde as the starting material according to the published route (Li *et al.*, 2010). The single crystals were obtained by recrystallization from the mixture of methanol and dichloromethane at room temperature.

### 3. Refinement

All H atoms were located in a difference map but placed in geometrically idealized positions and constrained to ride on their parent atoms with C—H distance of 0.93 Å, and with *U*_iso_(H)=1.2U_iso_(C).

### Crystal data

**Table:**

C_22_H_12_N_4_O_2_	*F*(000) = 752
*M**_r_* = 364.36	*D*_x_ = 1.449 Mg m^−^^3^
Orthorhombic, *P**b**c**n*	Mo *K*α radiation, λ = 0.71073 Å
Hall symbol: -P 2n 2ab	Cell parameters from 6163 reflections
*a* = 7.0994 (14) Å	θ = 3.3–27.5°
*b* = 25.014 (5) Å	µ = 0.10 mm^−^^1^
*c* = 9.4083 (19) Å	*T* = 293 K
*V* = 1670.8 (6) Å^3^	Block, yellow
*Z* = 4	0.26 × 0.21 × 0.18 mm

### Data collection

**Table:**

Bruker APEXII CCD diffractometer	1565 independent reflections
Radiation source: fine-focus sealed tube	1382 reflections with *I* > 2σ(*I*)
Graphite monochromator	*R*_int_ = 0.035
φ and ω scans	θ_max_ = 25.5°, θ_min_ = 3.3°
Absorption correction: multi-scan (*SADABS*; Sheldrick, 2008*a*)	*h* = −8→8
*T*_min_ = 0.975, *T*_max_ = 0.983	*k* = −21→30
7791 measured reflections	*l* = −11→11

### Refinement

**Table:**

Refinement on *F*^2^	Primary atom site location: structure-invariant direct methods
Least-squares matrix: full	Secondary atom site location: difference Fourier map
*R*[*F*^2^ > 2σ(*F*^2^)] = 0.051	Hydrogen site location: inferred from neighbouring sites
*wR*(*F*^2^) = 0.114	H-atom parameters constrained
*S* = 1.18	*w* = 1/[σ^2^(*F*_o_^2^) + (0.050*P*)^2^ + 0.3579*P*] where *P* = (*F*_o_^2^ + 2*F*_c_^2^)/3
1565 reflections	(Δ/σ)_max_ < 0.001
127 parameters	Δρ_max_ = 0.16 e Å^−^^3^
0 restraints	Δρ_min_ = −0.19 e Å^−^^3^

### Special details

**Table:**

Geometry. All e.s.d.'s (except the e.s.d. in the dihedral angle between two l.s. planes) are estimated using the full covariance matrix. The cell e.s.d.'s are taken into account individually in the estimation of e.s.d.'s in distances, angles and torsion angles; correlations between e.s.d.'s in cell parameters are only used when they are defined by crystal symmetry. An approximate (isotropic) treatment of cell e.s.d.'s is used for estimating e.s.d.'s involving l.s. planes.
Refinement. Refinement of *F*^2^ against ALL reflections. The weighted *R*-factor *wR* and goodness of fit *S* are based on *F*^2^, conventional *R*-factors *R* are based on *F*, with *F* set to zero for negative *F*^2^. The threshold expression of *F*^2^ > σ(*F*^2^) is used only for calculating *R*-factors(gt) *etc*. and is not relevant to the choice of reflections for refinement. *R*-factors based on *F*^2^ are statistically about twice as large as those based on *F*, and *R*- factors based on ALL data will be even larger.

### Fractional atomic coordinates and isotropic or equivalent isotropic displacement parameters (Å^2^)

**Table:**

	*x*	*y*	*z*	*U*_iso_*/*U*_eq_	
N1	0.14263 (19)	0.43286 (5)	0.35255 (15)	0.0303 (4)	
C6	0.0687 (2)	0.47894 (6)	0.30306 (18)	0.0284 (4)	
C2	0.1413 (2)	0.52917 (6)	0.35808 (18)	0.0297 (4)	
O1	0.19388 (17)	0.29767 (4)	0.25670 (14)	0.0391 (4)	
C3	0.2798 (2)	0.53071 (7)	0.46419 (19)	0.0337 (4)	
H3	0.3250	0.4992	0.5038	0.040*	
C7	0.0756 (2)	0.38724 (6)	0.30014 (17)	0.0295 (4)	
C1	0.0746 (2)	0.57801 (6)	0.30375 (18)	0.0302 (4)	
N2	0.1436 (2)	0.62587 (6)	0.34706 (16)	0.0373 (4)	
C8	0.1713 (2)	0.33906 (6)	0.35072 (18)	0.0307 (4)	
C9	0.2526 (3)	0.32685 (7)	0.4760 (2)	0.0377 (5)	
H9	0.2574	0.3483	0.5566	0.045*	
C4	0.3478 (3)	0.57901 (7)	0.5089 (2)	0.0394 (5)	
H4	0.4396	0.5810	0.5792	0.047*	
C5	0.2760 (3)	0.62534 (7)	0.4462 (2)	0.0408 (5)	
H5	0.3244	0.6580	0.4762	0.049*	
C11	0.2910 (3)	0.25895 (7)	0.3286 (2)	0.0401 (5)	
H11	0.3254	0.2261	0.2904	0.048*	
C10	0.3295 (3)	0.27471 (7)	0.4608 (2)	0.0401 (5)	
H10	0.3942	0.2553	0.5296	0.048*	

### Atomic displacement parameters (Å^2^)

**Table:**

	*U*^11^	*U*^22^	*U*^33^	*U*^12^	*U*^13^	*U*^23^
N1	0.0335 (8)	0.0231 (7)	0.0344 (8)	0.0012 (6)	−0.0021 (6)	−0.0002 (6)
C6	0.0291 (8)	0.0233 (8)	0.0329 (9)	0.0000 (7)	0.0015 (7)	−0.0003 (7)
C2	0.0295 (8)	0.0248 (9)	0.0348 (9)	0.0002 (6)	0.0006 (7)	−0.0028 (7)
O1	0.0482 (7)	0.0258 (6)	0.0433 (7)	0.0083 (5)	−0.0033 (6)	−0.0036 (5)
C3	0.0337 (9)	0.0267 (9)	0.0409 (10)	0.0023 (7)	−0.0052 (8)	−0.0012 (7)
C7	0.0329 (8)	0.0243 (8)	0.0313 (9)	0.0006 (7)	0.0007 (7)	−0.0006 (7)
C1	0.0317 (8)	0.0240 (8)	0.0348 (9)	−0.0012 (7)	0.0022 (7)	−0.0021 (7)
N2	0.0404 (8)	0.0249 (8)	0.0465 (9)	−0.0024 (6)	−0.0053 (7)	−0.0025 (6)
C8	0.0335 (9)	0.0205 (8)	0.0380 (10)	−0.0008 (6)	0.0003 (8)	−0.0012 (7)
C9	0.0423 (10)	0.0297 (10)	0.0412 (10)	0.0029 (8)	−0.0065 (9)	0.0010 (8)
C4	0.0378 (10)	0.0339 (10)	0.0465 (11)	−0.0014 (8)	−0.0097 (8)	−0.0058 (8)
C5	0.0434 (10)	0.0268 (10)	0.0523 (12)	−0.0044 (8)	−0.0067 (9)	−0.0072 (8)
C11	0.0407 (10)	0.0232 (9)	0.0564 (12)	0.0065 (8)	0.0031 (9)	0.0041 (8)
C10	0.0380 (10)	0.0334 (11)	0.0490 (12)	0.0056 (8)	−0.0017 (9)	0.0111 (9)

### Geometric parameters (Å, º)

**Table:**

N1—C7	1.331 (2)	C1—N2	1.356 (2)
N1—C6	1.349 (2)	C1—C1^i^	1.465 (3)
C6—C6^i^	1.396 (3)	N2—C5	1.325 (2)
C6—C2	1.453 (2)	C8—C9	1.347 (2)
C2—C3	1.402 (2)	C9—C10	1.421 (2)
C2—C1	1.406 (2)	C9—H9	0.9300
O1—C11	1.368 (2)	C4—C5	1.396 (3)
O1—C8	1.371 (2)	C4—H4	0.9300
C3—C4	1.367 (2)	C5—H5	0.9300
C3—H3	0.9300	C11—C10	1.333 (3)
C7—C7^i^	1.429 (3)	C11—H11	0.9300
C7—C8	1.463 (2)	C10—H10	0.9300
			
C7—N1—C6	117.74 (15)	C9—C8—O1	110.07 (15)
N1—C6—C6^i^	121.24 (9)	C9—C8—C7	132.06 (16)
N1—C6—C2	118.53 (16)	O1—C8—C7	117.81 (14)
C6^i^—C6—C2	120.17 (10)	C8—C9—C10	106.51 (16)
C3—C2—C1	118.10 (15)	C8—C9—H9	126.7
C3—C2—C6	121.75 (15)	C10—C9—H9	126.7
C1—C2—C6	120.14 (16)	C3—C4—C5	118.34 (17)
C11—O1—C8	105.93 (14)	C3—C4—H4	120.8
C4—C3—C2	119.40 (16)	C5—C4—H4	120.8
C4—C3—H3	120.3	N2—C5—C4	124.37 (16)
C2—C3—H3	120.3	N2—C5—H5	117.8
N1—C7—C7^i^	120.87 (9)	C4—C5—H5	117.8
N1—C7—C8	114.83 (15)	C10—C11—O1	110.82 (16)
C7^i^—C7—C8	124.29 (9)	C10—C11—H11	124.6
N2—C1—C2	122.43 (16)	O1—C11—H11	124.6
N2—C1—C1^i^	117.94 (10)	C11—C10—C9	106.66 (16)
C2—C1—C1^i^	119.63 (10)	C11—C10—H10	126.7
C5—N2—C1	117.32 (15)	C9—C10—H10	126.7
			
C7—N1—C6—C6^i^	−2.2 (3)	C1^i^—C1—N2—C5	178.75 (19)
C7—N1—C6—C2	−179.43 (14)	C11—O1—C8—C9	0.52 (19)
N1—C6—C2—C3	−2.5 (3)	C11—O1—C8—C7	178.19 (15)
C6^i^—C6—C2—C3	−179.73 (19)	N1—C7—C8—C9	33.1 (3)
N1—C6—C2—C1	176.70 (15)	C7^i^—C7—C8—C9	−148.3 (2)
C6^i^—C6—C2—C1	−0.5 (3)	N1—C7—C8—O1	−143.91 (15)
C1—C2—C3—C4	−1.4 (3)	C7^i^—C7—C8—O1	34.7 (3)
C6—C2—C3—C4	177.83 (17)	O1—C8—C9—C10	−0.5 (2)
C6—N1—C7—C7^i^	−2.9 (3)	C7—C8—C9—C10	−177.69 (18)
C6—N1—C7—C8	175.76 (14)	C2—C3—C4—C5	−0.1 (3)
C3—C2—C1—N2	2.1 (3)	C1—N2—C5—C4	−0.3 (3)
C6—C2—C1—N2	−177.09 (15)	C3—C4—C5—N2	1.0 (3)
C3—C2—C1—C1^i^	−177.92 (18)	C8—O1—C11—C10	−0.4 (2)
C6—C2—C1—C1^i^	2.9 (3)	O1—C11—C10—C9	0.1 (2)
C2—C1—N2—C5	−1.3 (3)	C8—C9—C10—C11	0.2 (2)

Symmetry code: (i) −*x*, *y*, −*z*+1/2.
